# (*E*)-4-(2-Chloro-1-hy­droxy-2,6,6-tri­methyl­cyclo­hex­yl)but-3-en-2-one

**DOI:** 10.1107/S1600536812048544

**Published:** 2012-11-30

**Authors:** Shan Liu, Xiao-Yan Yang, Yu-Ling Zhang

**Affiliations:** a Nanjing College of Chemical Technology, No. 625 Geguan Road, Luhe, Nanjing 210048, People’s Republic of China

## Abstract

In the title mol­ecule, C_13_H_21_ClO_2_, there is an intra­molecular C—H⋯Cl hydrogen bond. The conformation about the C=C bond is *E* and the six-membered ring has a chair conformation. In the crystal, mol­ecules are linked by pairs of O—H⋯O hydrogen bonds, forming inversion dimers, which are consolidated by C—H⋯O hydrogen bonds. The dimers are linked *via* C—H.·O hydrogen bonds, forming chains along [100].

## Related literature

For the use of (*E*)-4-(2-chloro-1-hy­droxy-2,6,6-trimethyl­cyclo­hex­yl)but-3-en-2-one, see: Sakai *et al.* (1992 ▶). For bond-length data, see: Allen *et al.* (1987 ▶).
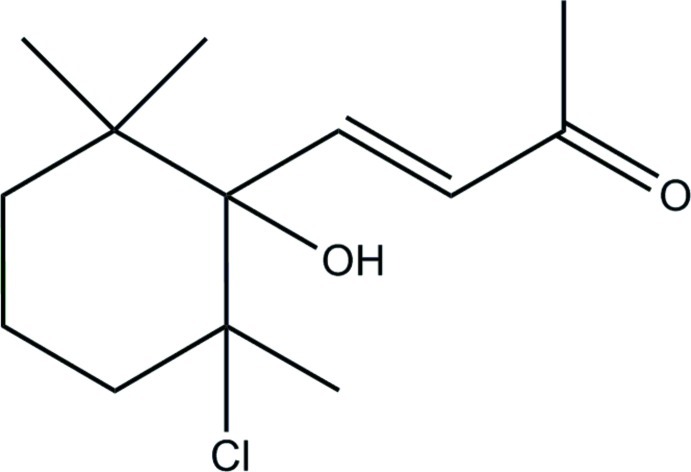


## Experimental

### 

#### Crystal data

C_13_H_21_ClO_2_
*M* *_r_* = 244.75
Monoclinic, 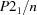

*a* = 6.266 (1) Å
*b* = 8.586 (2) Å
*c* = 24.868 (5) Å
β = 92.24 (3)°
*V* = 1336.9 (5) Å^3^
*Z* = 4
Mo *K*α radiation
μ = 0.27 mm^−1^
*T* = 298 K
0.30 × 0.20 × 0.10 mm

#### Data collection

Enraf–Nonius CAD-4 diffractometer
Absorption correction: ψ scan (North *et al.*, 1968 ▶) *T*_min_ = 0.923, *T*_max_ = 0.973
2688 measured reflections
2450 independent reflections
1611 reflections with *I* > 2σ(*I*)
*R*_int_ = 0.068
3 standard reflections every 200 reflections intensity decay: 1%

#### Refinement

*R*[*F* ^2^ > 2σ(*F* ^2^)] = 0.068
*wR*(*F* ^2^) = 0.179
*S* = 1.00
2450 reflections
145 parameters
1 restraint
H-atom parameters constrained
Δρ_max_ = 0.45 e Å^−3^
Δρ_min_ = −0.29 e Å^−3^

### 

Data collection: *CAD-4 Software* (Enraf–Nonius, 1985 ▶); cell refinement: *CAD-4 Software*; data reduction: *XCAD4* (Harms & Wocadlo, 1995 ▶); program(s) used to solve structure: *SHELXS97* (Sheldrick, 2008 ▶); program(s) used to refine structure: *SHELXL97* (Sheldrick, 2008 ▶); molecular graphics: *SHELXTL* (Sheldrick, 2008 ▶); software used to prepare material for publication: *SHELXTL*.

## Supplementary Material

Crystal structure: contains datablock(s) I, global. DOI: 10.1107/S1600536812048544/zj2098sup1.cif

Structure factors: contains datablock(s) I. DOI: 10.1107/S1600536812048544/zj2098Isup2.hkl

Supplementary material file. DOI: 10.1107/S1600536812048544/zj2098Isup3.cml

Additional supplementary materials:  crystallographic information; 3D view; checkCIF report

## Figures and Tables

**Table 1:** Hydrogen-bond geometry (Å, °)

*D*—H⋯*A*	*D*—H	H⋯*A*	*D*⋯*A*	*D*—H⋯*A*
O1—H1*A*⋯O2^i^	0.82	2.12	2.858 (3)	149
C7—H7*A*⋯O2^i^	0.96	2.58	3.473 (5)	155
C8—H8*C*⋯Cl	0.96	2.59	3.257 (3)	127
C13—H13*C*⋯O1^ii^	0.96	2.59	3.536 (4)	169

Symmetry codes: (i) 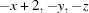; (ii) 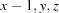.

**Fig. 1.**
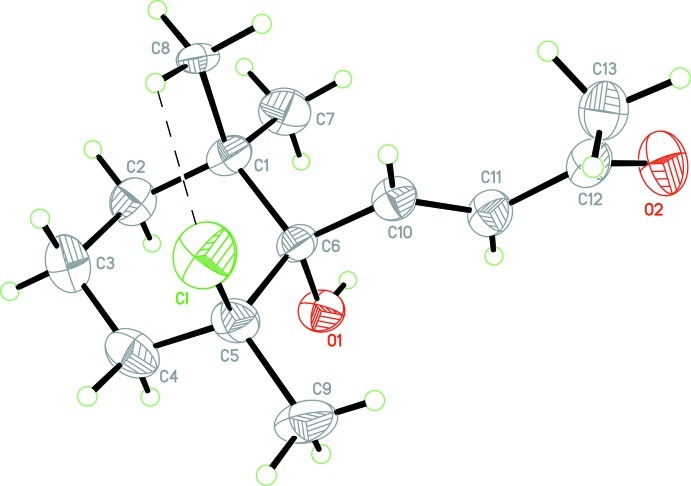
The molecular structure of the title compound with the atom numbering scheme. Displacement ellipsoids are drawn at the 40% probability level. H atoms are presented as small spheres of arbitrary radius.

**Fig. 2.**
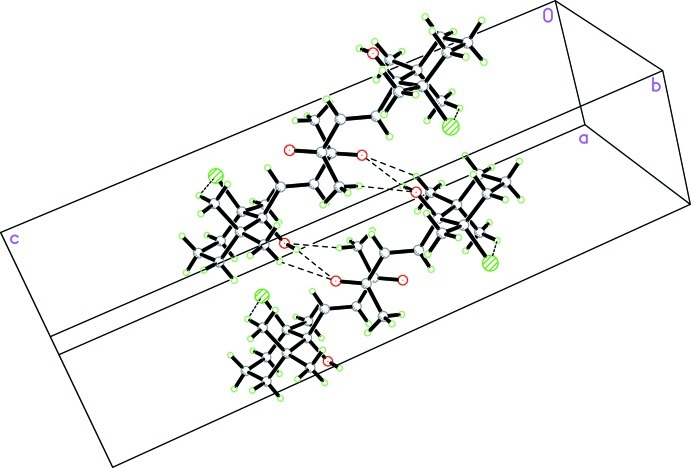
A view of the C—H···Cl, C—H···O and O—H···O hydrogen bonds (dotted lines) in the crystal structure of the title compound. [Symmter codes: (i) 2 - x,-y,-z; (ii) 2 - x,-y,-z; (iii) -1 + x,y,z.]

## supplementary crystallographic information

### Comment

(*E*)-4-(2-Chloro-1-hydroxy-2,6,6-trimethylcyclohexyl)but-3-en-2-one is an important intermediate used to synthesize abscisic acid (ABA), which has important activities as a plant hormone (Sakai, *et al.*, 1992). We report here the crystal structure of the title compound (Fig. 1).

In the title molecule, bond lengths (Allen *et al.*, 1987) and angles are within normal ranges. In the crystal packing (Fig. 2), mmolecules are linked to form three-dimensional framework by intra- and intermolecular C—H···Cl, C—H···O and O—H···O hydrogen bonds, which may be effective for the stabilization of the crystals (see, Table 1).

### Experimental

(*E*)-4-(2-Chloro-1-hydroxy-2,6,6-trimethylcyclohexyl)but-3-en-2-one was prepared by the reaction of (*E*)-4-(2,2,6-trimethyl-7- oxabicyclo[4.1.0]heptan-1-yl)but-3-en-2-one (20.8 g, 0.100 mmol) and 1*M* hydrochloric acid (30 ml) in ethanol (150 ml) at 273 K for 3 h, and separated by column chromatography on silica gel (hexane / ethyl acetate = 8/2, V/V) with a yield of 50%. Single crystals were obtained by dissolving the title compound (0.50 g, 2.04 mmol) in ethyl acetate (30 ml) and evaporating the solvent slowly at 288–293 K for about 1 d.

### Refinement

H atoms were positioned geometrically, with O—H = 0.82, and constrained to ride on their parent atoms, with *U*_iso_(H) = *xU*_eq_(C/O), where *x* = 1.5 for H.

### Crystal data

**Table:**

C_13_H_21_ClO_2_	*F*(000) = 528
*M**_r_* = 244.75	*D*_x_ = 1.216 Mg m^−^^3^
Monoclinic, *P*2_1_/*n*	Melting point = 380–383 K
Hall symbol: -P 2yn	Mo *K*α radiation, λ = 0.71073 Å
*a* = 6.266 (1) Å	Cell parameters from 25 reflections
*b* = 8.586 (2) Å	θ = 10–13°
*c* = 24.868 (5) Å	µ = 0.27 mm^−^^1^
β = 92.24 (3)°	*T* = 298 K
*V* = 1336.9 (5) Å^3^	Cube, colorless
*Z* = 4	0.30 × 0.20 × 0.10 mm

### Data collection

**Table:**

Enraf–Nonius CAD-4 diffractometer	1611 reflections with *I* > 2σ(*I*)
Radiation source: fine-focus sealed tube	*R*_int_ = 0.068
Graphite monochromator	θ_max_ = 25.4°, θ_min_ = 1.6°
ω/2θ scans	*h* = 0→7
Absorption correction: ψ scan (North *et al.*, 1968)	*k* = 0→10
*T*_min_ = 0.923, *T*_max_ = 0.973	*l* = −29→29
2688 measured reflections	3 standard reflections every 200 reflections
2450 independent reflections	intensity decay: 1%

### Refinement

**Table:**

Refinement on *F*^2^	Primary atom site location: structure-invariant direct methods
Least-squares matrix: full	Secondary atom site location: difference Fourier map
*R*[*F*^2^ > 2σ(*F*^2^)] = 0.068	Hydrogen site location: inferred from neighbouring sites
*wR*(*F*^2^) = 0.179	H-atom parameters constrained
*S* = 1.00	*w* = 1/[σ^2^(*F*_o_^2^) + (0.1*P*)^2^ + 0.3*P*] where *P* = (*F*_o_^2^ + 2*F*_c_^2^)/3
2450 reflections	(Δ/σ)_max_ < 0.001
145 parameters	Δρ_max_ = 0.45 e Å^−^^3^
1 restraint	Δρ_min_ = −0.29 e Å^−^^3^

### Special details

**Table:**

Geometry. All e.s.d.'s (except the e.s.d. in the dihedral angle between two l.s. planes) are estimated using the full covariance matrix. The cell e.s.d.'s are taken into account individually in the estimation of e.s.d.'s in distances, angles and torsion angles; correlations between e.s.d.'s in cell parameters are only used when they are defined by crystal symmetry. An approximate (isotropic) treatment of cell e.s.d.'s is used for estimating e.s.d.'s involving l.s. planes.
Refinement. Refinement of *F*^2^ against ALL reflections. The weighted *R*-factor *wR* and goodness of fit *S* are based on *F*^2^, conventional *R*-factors *R* are based on *F*, with *F* set to zero for negative *F*^2^. The threshold expression of *F*^2^ > σ(*F*^2^) is used only for calculating *R*-factors(gt) *etc*. and is not relevant to the choice of reflections for refinement. *R*-factors based on *F*^2^ are statistically about twice as large as those based on *F*, and *R*- factors based on ALL data will be even larger.

### Fractional atomic coordinates and isotropic or equivalent isotropic displacement parameters (Å^2^)

**Table:**

	*x*	*y*	*z*	*U*_iso_*/*U*_eq_	
Cl	0.73792 (16)	0.51609 (11)	0.14841 (4)	0.0672 (4)	
O1	1.1529 (3)	0.2036 (2)	0.09894 (8)	0.0447 (6)	
H1A	1.1530	0.1117	0.0899	0.067*	
C1	0.8845 (5)	0.1320 (3)	0.16368 (12)	0.0425 (7)	
O2	0.6807 (4)	0.0827 (3)	−0.05952 (10)	0.0669 (8)	
C2	1.0445 (5)	0.1541 (4)	0.21162 (13)	0.0505 (8)	
H2A	1.0002	0.0904	0.2414	0.061*	
H2B	1.1839	0.1177	0.2015	0.061*	
C3	1.0635 (6)	0.3233 (4)	0.23029 (15)	0.0622 (10)	
H3A	0.9271	0.3583	0.2430	0.075*	
H3B	1.1681	0.3306	0.2600	0.075*	
C4	1.1304 (6)	0.4266 (4)	0.18449 (15)	0.0549 (9)	
H4A	1.1366	0.5335	0.1972	0.066*	
H4B	1.2733	0.3972	0.1748	0.066*	
C5	0.9854 (5)	0.4199 (3)	0.13459 (13)	0.0433 (8)	
C6	0.9465 (5)	0.2448 (3)	0.11647 (11)	0.0365 (7)	
C7	0.8980 (6)	−0.0372 (4)	0.14485 (15)	0.0601 (10)	
H7A	1.0381	−0.0573	0.1321	0.090*	
H7B	0.7935	−0.0550	0.1162	0.090*	
H7C	0.8710	−0.1056	0.1744	0.090*	
C8	0.6432 (4)	0.1588 (3)	0.18449 (11)	0.0300 (6)	
H8A	0.6166	0.0868	0.2130	0.045*	
H8B	0.5415	0.1418	0.1552	0.045*	
H8C	0.6298	0.2635	0.1975	0.045*	
C9	1.0807 (6)	0.5136 (4)	0.08881 (15)	0.0615 (10)	
H9A	1.2142	0.4680	0.0795	0.092*	
H9B	1.1039	0.6192	0.1003	0.092*	
H9C	0.9836	0.5122	0.0580	0.092*	
C10	0.7826 (5)	0.2374 (3)	0.07141 (12)	0.0397 (7)	
H10A	0.6470	0.2752	0.0781	0.048*	
C11	0.8145 (5)	0.1811 (4)	0.02232 (12)	0.0447 (8)	
H11A	0.9515	0.1480	0.0148	0.054*	
C12	0.6479 (5)	0.1680 (3)	−0.02048 (12)	0.0447 (8)	
C13	0.4476 (5)	0.2548 (4)	−0.01817 (13)	0.0527 (9)	
H13A	0.3583	0.2309	−0.0493	0.079*	
H13B	0.4775	0.3645	−0.0174	0.079*	
H13C	0.3755	0.2260	0.0137	0.079*	

### Atomic displacement parameters (Å^2^)

**Table:**

	*U*^11^	*U*^22^	*U*^33^	*U*^12^	*U*^13^	*U*^23^
Cl	0.0756 (7)	0.0548 (6)	0.0719 (7)	0.0143 (5)	0.0108 (5)	−0.0036 (4)
O1	0.0380 (11)	0.0435 (12)	0.0534 (13)	0.0036 (9)	0.0116 (10)	−0.0076 (10)
C1	0.0447 (17)	0.0388 (16)	0.0450 (17)	0.0001 (14)	0.0132 (14)	0.0023 (14)
O2	0.0759 (18)	0.0694 (17)	0.0551 (14)	0.0135 (14)	0.0005 (13)	−0.0249 (13)
C2	0.0510 (19)	0.0507 (19)	0.0496 (19)	0.0063 (16)	−0.0001 (16)	0.0062 (16)
C3	0.071 (2)	0.064 (2)	0.051 (2)	0.000 (2)	−0.0090 (18)	−0.0096 (18)
C4	0.051 (2)	0.0417 (18)	0.071 (2)	−0.0013 (15)	−0.0016 (18)	−0.0108 (17)
C5	0.0494 (19)	0.0305 (15)	0.0503 (18)	−0.0027 (14)	0.0056 (15)	−0.0040 (13)
C6	0.0393 (16)	0.0359 (15)	0.0350 (15)	−0.0037 (12)	0.0098 (13)	−0.0003 (12)
C7	0.078 (3)	0.0330 (17)	0.070 (2)	−0.0073 (17)	0.006 (2)	0.0025 (16)
C8	0.0208 (12)	0.0345 (14)	0.0346 (14)	−0.0033 (11)	0.0010 (11)	0.0130 (11)
C9	0.070 (2)	0.0427 (19)	0.074 (2)	−0.0124 (17)	0.023 (2)	0.0067 (17)
C10	0.0392 (16)	0.0382 (16)	0.0423 (16)	0.0047 (13)	0.0072 (14)	−0.0021 (13)
C11	0.0503 (18)	0.0415 (17)	0.0429 (17)	0.0026 (14)	0.0077 (14)	−0.0041 (14)
C12	0.062 (2)	0.0335 (16)	0.0393 (16)	−0.0003 (14)	0.0105 (15)	−0.0040 (13)
C13	0.061 (2)	0.052 (2)	0.0450 (18)	0.0059 (17)	0.0027 (16)	−0.0053 (16)

### Geometric parameters (Å, º)

**Table:**

Cl—C5	1.802 (3)	C6—C10	1.491 (4)
O1—C6	1.425 (3)	C7—H7A	0.9600
O1—H1A	0.8200	C7—H7B	0.9600
C1—C7	1.530 (4)	C7—H7C	0.9600
C1—C2	1.539 (4)	C8—H8A	0.9600
C1—C6	1.582 (4)	C8—H8B	0.9600
C1—C8	1.633 (4)	C8—H8C	0.9600
O2—C12	1.240 (4)	C9—H9A	0.9600
C2—C3	1.528 (5)	C9—H9B	0.9600
C2—H2A	0.9700	C9—H9C	0.9600
C2—H2B	0.9700	C10—C11	1.336 (4)
C3—C4	1.516 (5)	C10—H10A	0.9300
C3—H3A	0.9700	C11—C12	1.466 (4)
C3—H3B	0.9700	C11—H11A	0.9300
C4—C5	1.510 (5)	C12—C13	1.463 (4)
C4—H4A	0.9700	C13—H13A	0.9600
C4—H4B	0.9700	C13—H13B	0.9600
C5—C9	1.534 (4)	C13—H13C	0.9600
C5—C6	1.586 (4)		
			
C6—O1—H1A	109.5	C10—C6—C5	110.3 (2)
C7—C1—C2	108.2 (3)	C1—C6—C5	114.1 (2)
C7—C1—C6	109.6 (3)	C1—C7—H7A	109.5
C2—C1—C6	109.1 (2)	C1—C7—H7B	109.5
C7—C1—C8	107.1 (2)	H7A—C7—H7B	109.5
C2—C1—C8	108.7 (2)	C1—C7—H7C	109.5
C6—C1—C8	114.0 (2)	H7A—C7—H7C	109.5
C3—C2—C1	113.1 (3)	H7B—C7—H7C	109.5
C3—C2—H2A	109.0	C1—C8—H8A	109.5
C1—C2—H2A	109.0	C1—C8—H8B	109.5
C3—C2—H2B	109.0	H8A—C8—H8B	109.5
C1—C2—H2B	109.0	C1—C8—H8C	109.5
H2A—C2—H2B	107.8	H8A—C8—H8C	109.5
C4—C3—C2	110.4 (3)	H8B—C8—H8C	109.5
C4—C3—H3A	109.6	C5—C9—H9A	109.5
C2—C3—H3A	109.6	C5—C9—H9B	109.5
C4—C3—H3B	109.6	H9A—C9—H9B	109.5
C2—C3—H3B	109.6	C5—C9—H9C	109.5
H3A—C3—H3B	108.1	H9A—C9—H9C	109.5
C5—C4—C3	114.8 (3)	H9B—C9—H9C	109.5
C5—C4—H4A	108.6	C11—C10—C6	125.4 (3)
C3—C4—H4A	108.6	C11—C10—H10A	117.3
C5—C4—H4B	108.6	C6—C10—H10A	117.3
C3—C4—H4B	108.6	C10—C11—C12	124.3 (3)
H4A—C4—H4B	107.5	C10—C11—H11A	117.8
C4—C5—C9	110.5 (3)	C12—C11—H11A	117.8
C4—C5—C6	110.5 (3)	O2—C12—C13	120.0 (3)
C9—C5—C6	110.2 (2)	O2—C12—C11	118.6 (3)
C4—C5—Cl	108.7 (2)	C13—C12—C11	121.4 (3)
C9—C5—Cl	105.3 (2)	C12—C13—H13A	109.5
C6—C5—Cl	111.4 (2)	C12—C13—H13B	109.5
O1—C6—C10	111.5 (2)	H13A—C13—H13B	109.5
O1—C6—C1	109.1 (2)	C12—C13—H13C	109.5
C10—C6—C1	110.5 (2)	H13A—C13—H13C	109.5
O1—C6—C5	101.0 (2)	H13B—C13—H13C	109.5
			
C7—C1—C2—C3	173.8 (3)	C8—C1—C6—C5	72.1 (3)
C6—C1—C2—C3	54.6 (4)	C4—C5—C6—O1	−69.0 (3)
C8—C1—C2—C3	−70.3 (3)	C9—C5—C6—O1	53.6 (3)
C1—C2—C3—C4	−58.1 (4)	Cl—C5—C6—O1	170.10 (19)
C2—C3—C4—C5	56.5 (4)	C4—C5—C6—C10	173.0 (2)
C3—C4—C5—C9	−173.5 (3)	C9—C5—C6—C10	−64.5 (3)
C3—C4—C5—C6	−51.2 (4)	Cl—C5—C6—C10	52.1 (3)
C3—C4—C5—Cl	71.4 (3)	C4—C5—C6—C1	48.0 (3)
C7—C1—C6—O1	−55.7 (3)	C9—C5—C6—C1	170.5 (3)
C2—C1—C6—O1	62.6 (3)	Cl—C5—C6—C1	−73.0 (3)
C8—C1—C6—O1	−175.7 (2)	O1—C6—C10—C11	8.0 (4)
C7—C1—C6—C10	67.2 (3)	C1—C6—C10—C11	−113.5 (3)
C2—C1—C6—C10	−174.5 (2)	C5—C6—C10—C11	119.4 (3)
C8—C1—C6—C10	−52.9 (3)	C6—C10—C11—C12	176.8 (3)
C7—C1—C6—C5	−167.9 (3)	C10—C11—C12—O2	−163.5 (3)
C2—C1—C6—C5	−49.6 (3)	C10—C11—C12—C13	17.4 (5)

### Hydrogen-bond geometry (Å, º)

**Table:**

*D*—H···*A*	*D*—H	H···*A*	*D*···*A*	*D*—H···*A*
O1—H1*A*···O2^i^	0.82	2.12	2.858 (3)	149
C7—H7*A*···O2^i^	0.96	2.58	3.473 (5)	155
C8—H8*C*···Cl	0.96	2.59	3.257 (3)	127
C13—H13*C*···O1^ii^	0.96	2.59	3.536 (4)	169

Symmetry codes: (i) −*x*+2, −*y*, −*z*; (ii) *x*−1, *y*, *z*.
